# Determining if Telehealth Can Reduce Health System Costs: Scoping Review

**DOI:** 10.2196/17298

**Published:** 2020-10-19

**Authors:** Centaine L Snoswell, Monica L Taylor, Tracy A Comans, Anthony C Smith, Leonard C Gray, Liam J Caffery

**Affiliations:** 1 Centre for Online Health The University of Queensland Brisbane Australia; 2 Centre for Health Services Research The University of Queensland Brisbane Australia; 3 Centre for Innovative Medical Technology University of Southern Denmark Odense Denmark

**Keywords:** cost-benefit analysis, telemedicine, review

## Abstract

**Background:**

Telehealth represents an opportunity for Australia to harness the power of technology to redesign the way health care is delivered. The potential benefits of telehealth include increased accessibility to care, productivity gains for health providers and patients through reduced travel, potential for cost savings, and an opportunity to develop culturally appropriate services that are more sensitive to the needs of special populations. The uptake of telehealth has been hindered at times by clinician reluctance and policies that preclude metropolitan populations from accessing telehealth services.

**Objective:**

This study aims to investigate if telehealth reduces health system costs compared with traditional service models and to identify the scenarios in which cost savings can be realized.

**Methods:**

A scoping review was undertaken to meet the study aims. Initially, literature searches were conducted using broad terms for telehealth and economics to identify economic evaluation literature in telehealth. The investigators then conducted an expert focus group to identify domains where telehealth could reduce health system costs, followed by targeted literature searches for corresponding evidence.

**Results:**

The cost analyses reviewed provided evidence that telehealth reduced costs when health system–funded travel was prevented and when telehealth mitigated the need for expensive procedural or specialist follow-up by providing competent care in a more efficient way. The expert focus group identified 4 areas of potential savings from telehealth: productivity gains, reductions in secondary care, alternate funding models, and telementoring. Telehealth demonstrated great potential for productivity gains arising from health system redesign; however, under the Australian activity-based funding, it is unlikely that these gains will result in cost savings. Secondary care use mitigation is an area of promise for telehealth; however, many studies have not demonstrated overall cost savings due to the cost of administering and monitoring telehealth systems. Alternate funding models from telehealth systems have the potential to save the health system money in situations where the consumers pay out of pocket to receive services. Telementoring has had minimal economic evaluation; however, in the long term it is likely to result in inadvertent cost savings through the upskilling of generalist and allied health clinicians.

**Conclusions:**

Health services considering implementing telehealth should be motivated by benefits other than cost reduction. The available evidence has indicated that although telehealth provides overwhelmingly positive patient benefits and increases productivity for many services, current evidence suggests that it does not routinely reduce the cost of care delivery for the health system.

## Introduction

The sustainability of health systems is a major concern for governments worldwide. The financial viability of the health system is of particular concern, in light of both increasing costs and the increasing ratio of health expenditure to gross domestic product (GDP). For example, in Australia, in the decade to 2017, health care expenditure nearly doubled and the ratio of health expenditure to GDP increased from 8.75% to 10.28% [1]. Similarly, in the United States during the same time frame, health expenditure grew by 50% and the ratio of health expenditure to GDP increased from 15.9% to 17.9% [2]. This has catalyzed an imperative to reduce the cost of providing health care, as these increases are not sustainable long term.

Telehealth is the delivery of clinical health services using information and communication technologies to bridge the geographic separation of the clinician and consumer. Telehealth could potentially impact costs due to shorter interactions, reduced travel, economies of scale, increased revenues, or moving elements of care from clinicians to technology (eg, monitoring device) or to the patient themselves. The potential to reduce the cost of health care is one of the predominant reasons for the interest in implementing telehealth, followed closely by a desire to improve access to health care. Telehealth is often used to substitute a proportion of in-person encounters, and this substitution raises the question of a relative cost reduction.

Demonstrated cost-effective interventions may not be implemented due to budget constraints [3]. Hence, many health care organizations may be limited to implementing only telehealth interventions where cost reduction can be realized within a budgetary cycle and there is no cost increase associated with implementation. However, these programs are few, and those that start successfully are not always sustainable in the long run, scalable, or transferable to other settings. Although proponents of telehealth often have projected health care savings, there is a dearth of evidence to support this view. Important questions relating to cost and sustainability remain unanswered [4].

Reviews of telehealth cost-effectiveness are often limited to one clinical specialty, service modality, or country [5-8]. Limited research has looked collectively at available evidence, and to our knowledge, none have synthesized it from the perspective of the health system. Despite the differences between international health systems, identifying and collating information regarding the cost-saving potential of telehealth is valuable.

The aims of this scoping review were twofold. First, to investigate if telehealth reduces health system costs compared with traditional service models using international evidence, and second, to identify the scenarios in which cost savings can be realized.

## Methods

### Study Design

**Figure 1:**
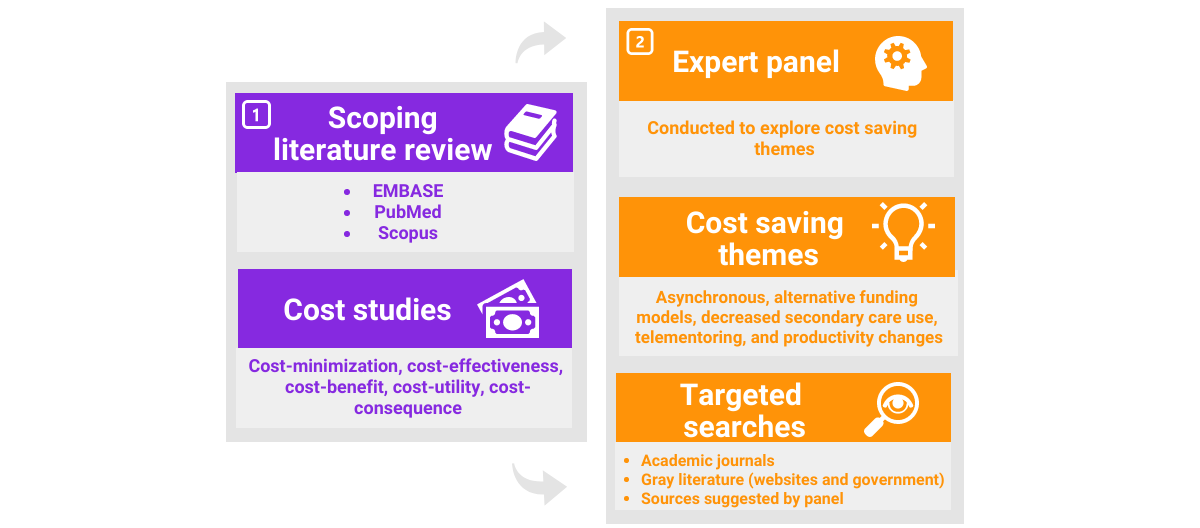
Scoping review methods.

The Arksey and O’Malley scoping review method was used to achieve the aims of this study [9]. This methodology involved an initial literature review, followed by an expert focus group, and finally targeted literature searches based on the focus group discussion (Figure 1) [9]. A scoping review method was chosen due to the volume of literature [10]. Reporting of the methods and results was performed in accordance with the Preferred Reporting Items for Systematic reviews and Meta-Analyses extension for Scoping Reviews (PRISMA-ScR) Checklist [11].

### Initial Literature Review (Economic Evidence)

To identify literature reporting the results of economic evaluations in telehealth, initial literature searches were conducted using terms relating to telehealth, telemedicine, and economics. Searches were conducted using the PubMed, EMBASE, and Scopus databases. Search results were restricted to publications available in English, and no restriction was placed on the country of origin or health system model described. The scope of the review was restricted to short- to mid-term (6 months to 3 years) term and cost implications reported from the perspective of the health system payer (ie, health service or hospital). After the completion of database searches, duplicates were removed, titles and abstracts were screened by 2 authors (MT and CS), and a full-text review of articles was conducted by 3 authors (MT, LC, and CS). Included studies were categorized as cost-minimization analysis (CMA), cost-effectiveness analysis (CEA), and cost-utility analysis (CUA) studies [6,12,13]. Further descriptions of these categories are provided in Table 1.

**Table 1:** Description of cost analysis types.

Method	Description
CMA^a^ [14]	CMA requires either proof or a stated assumption that the two comparators are equally effective, and therefore, the analysis only examines the difference in cost between the comparators. When comparing CMA, it is important to examine the items included for costing for each comparator as well as the final reported result.
CEA^b^ [12]	CEA quantifies both the costs and a measurable effect (eg, blood pressure in mm Hg or days to diagnosis) from the comparators and presents them as a cost per increment of effectiveness. Due to the variety in measured effects, CEA are not easily comparable unless they use the same measure for effectiveness.
CUA^c^	CUA uses measures of cost and health-related quality of life (often expressed as a utility value) to compare interventions with usual care. Although more comparable, it is important to examine not only the cost estimations but also the method of eliciting health-related quality of life within each study.

^a^CMA: cost-minimization analysis.

^b^CEA: cost-effectiveness analysis.

^c^CUA: cost-utility analysis.

Data were extracted from the included studies by two investigators, and any disagreements in data extraction were discussed until a consensus was reached. The results were synthesized, and descriptions of systems in which telehealth reduces costs to the health system were identified and reported. All articles were quality assessed using the Consolidated Health Economic Evaluation Reporting Standards (CHEERS) [15]. CHEERS is a 24-point quality assessment tool for published economic evaluations. It includes domains to assess the reporting of relevant economic principles such as currency, discounting, time horizon, effectiveness measures, choice of outcome, assumptions, and model choice. The CMA studies were assessed according to 20 of the 24 points in the checklist; choice of health outcome, measurement of effectiveness, measurement and valuation of preference-based outcomes, and incremental costs and outcomes were not applicable. All costs were converted to 2019 US $ to allow for ease of comparison. The synthesis of the findings was narrative.

### Expert Panel Focus Group

Ethics approval to undertake the focus group was received from The University of Queensland’s Human Research Ethics Committee (Approval # 2018002428).

The framework proposed by Arksey and O’Malley [9] identifies that reviews can be enhanced by including a consultation process. To this end, investigators conducted an expert focus group to identify the domains where telehealth could reduce health system costs.

National experts in telehealth and economics were sent an invitation to attend the focus group on “How can telehealth reduce health system costs?”. Experts were identified and recruited through existing relationships between the research team and the Australasian Telehealth Society who assisted with recommending individuals with both health economics and telehealth expertise. A total of 16 experts were invited to participate and 9 agreed to represent 7 different organizations. Participation was via focus group (n=7) or via email and telephone with the investigators (n=2).

Attendees were given the option to attend in person or via videoconference. Before attendance, invitees were emailed a discussion paper. Invitees were asked if they agreed or disagreed with the points in the discussion paper and if any additional factors should be considered. Some invitees who were unable to attend the meeting provided feedback via email or phone. Open-ended questions on the potential for cost savings arising from telehealth were used to facilitate discussion in the focus group. The discussion was recorded and notes were recorded. Topics suggested by the expert panel were synthesized and categorized under distinct topic headings (domains). These domains were used to direct further literature searches. By including the focus group consultative exercise where participants were given potential domains, it was possible to elicit more ideas on the subject matter, resulting in richer data [9].

### Targeted Literature Searches

Subsequent to the focus groups, further literature searches were conducted to locate evidence on domains identified by the expert panel. Literature searches were conducted using both broad search terms and domain-specific terms to identify supporting evidence. Searches were conducted in PubMed, EMBASE, Scopus, and gray literature. Additionally, members of the expert panel contributed relevant evidence items (designated as hand searches). No restrictions on publication date were set during any search process. Searches were conducted from May 2018 to January 2019. Articles were omitted when the telehealth modality referenced telephone only. Search terms included, but were not limited to, telehealth, telemedicine, store-and-forward and other telehealth nomenclature, and domain-specific search terms such as secondary care or productivity. No restrictions were placed on the country of origin or the health system described in the analyses.

The level of evidence was described using the National Health and Medical Research Council guidelines [16], where, for example, I is a systematic review of randomized controlled trials (RCTs), II is an RCT, III is a comparative study with or without controls, and IV is a case series. The results of the subsequent articles identified through the targeted searches were summarized.

## Results

### CMA

**Figure 2:**
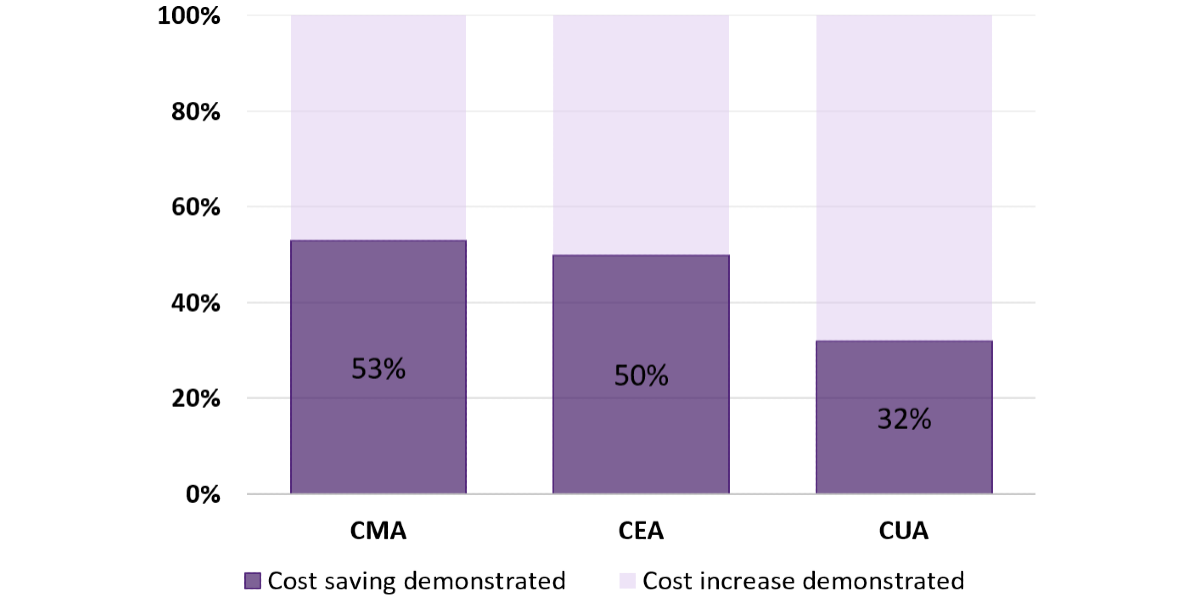
Proportion of studies identified that saved costs. CEA: cost-effectiveness analysis; CMA: cost-minimization analysis; CUA: cost-utility analysis.

Searches and screening identified 17 cost-minimization studies that reported their results from the perspective of the health system. Of these 17, 9 studies (53%) reported telehealth to be cost saving compared with conventional care (Figure 2), 6 studies (35%) reported telehealth to be cost saving after a workload threshold was achieved, and 2 studies (12%) reported telehealth to be more expensive than conventional care. The overall quality of reporting was sound, with an average score of 15 out of 20 (Table 2).

**Table 2:** Cost-minimization analysis demonstrating lower costs for telehealth from the perspective of the health system.

Reference	Telehealth modality and clinical focus	CHEERS^a^ score (out of 20)	Findings in US $ 2019	Initial investment in US $ 2019	Reason for lower cost in the telehealth group
Kovács et al (2017) [17], Hungary	Store-and-forward system for screening for retinopathy of prematurity	18	Cost per examination for telehealth was less than in-person examination.	$199,959.03 including equipment and implementation costs	Saved patient transport costs; saved working hours
Buysse et al (2008) [18], Belgium	Remote monitoring for high-risk pregnancy replacing extended hospital admissions	14	Cost reduction for remote monitoring of $233,958 per year.	$15,409.17	Saved admitted days. If an average of 8 patients were suitable for and accepted remote monitoring each month, an average of 14.7 admitted days could be replaced by remote monitoring
Xu et al (2008) [19], Australia	Videoconference for ear, nose and throat consultations	19	Costs of $108 per consultation for telehealth versus $155 for in-person consultation when caseload >100 consultations per year; saving realized despite a patient-end pediatrician cost to telehealth.	$31,509.38	Saved patient and family travel
Armstrong et al (2007) [20], United States	Store-and-forward system for dermatology screening, diagnosis, and triage	15	Teledermatology practice had an hourly operating cost of $361 versus $456 for conventional care	Not reported	When the patient-end is in a rural area (cheaper to rent space in those clinics)
Smith et al (2007) [21], Australia	Pediatric videoconference service for consultation reducing travel requirements	17	At caseload >774 cases/5 years telehealth is cost savings compared with in-person; $598,203 saved over 5 years.	Not reported	Saved patient transport costs
Pare et al (2006) [22], Canada	Remote monitoring for patients with chronic obstructive pulmonary disorder by nurses, in place of regular home visits	13	Telehealth realizes $361 in savings per patient or $8566 total service cost savings compared with traditional in-home care program over 6-months (~$13,713 per annum).	$24,609.38	Reduced home visits by nurses (saved travel) and reduced salary for home-visit nurses (increased productivity) and reduced hospitalizations (secondary care usage)
Labiris et al (2005) [23], Greece	Multispecialty videoconference consultations service (mainly orthopedics and dermatology) in place of in-person consultations	13	Cost per consultation for telehealth $327 versus $333 for conventional care.	$34,356.78	Saved transportation costs
Norum et al (2005) [24], Norway	Hybrid system for radiotherapy involving store-and-forward simulation planning and remote oncologist supervision via videoconference	15	At workload >9-12 patients, telehealth is less expensive when patient transport by air is required.	$112,115.99	Avoided emergency transfers
Scuffham et al (2002) [25], United Kingdom (Scotland)	Generalist dentist videoconference with specialist dentists from a metropolitan center reducing the need for travel by patients or specialists	19	Teledentistry ($233) is more expensive compared with outreach ($156) but less expensive when compared in-person care ($662) per patient treated.	Not reported	Saved patient travel costs/subsidy
Bjørvig et al (2002) [26], Norway	Store-and-forward system for diabetic retinopathy screening	17	At caseloads of >110, telehealth is cost saving. At workloads <110, telehealth is more expensive than conventional care; at very low workloads (n=20), telehealth is around 20 times more expensive than conventional care per consultation; at high workloads (n=200), telehealth costs around 67% of conventional care per consultation.	Not reported	Saved patient travel
Harno et al (2001) [27], Finland	Review and triage of orthopedic cases via videoconference	12	Telehealth was $3954 (total service cost) less expensive than the traditional referral model.	Not reported	Triage by VC^b^ decreased the number of in-person hospital visits
Bergmo et al (2000) [28], Norway	Store-and-forward system for dermatology screening, diagnosis, and triage	16	At caseload >195 patients per year, telehealth ($96,042.79) costs less than hybrid outreach/patient travel service as a whole ($179,634.98), patient travel ($333,568.03) or locally employed dermatologists ($81,355.24); actual workload was 375 patients.	$81355.24	Saved patient and/or clinician travel
Harno et al (2000) [29], Finland	Triage of specialist cases via email and/or videoconference	13	Telehealth is less expensive with saving of $10,874 over 8 months for the service.	Not reported	Triage by email and/or VC decreased the number of hospital visits
McCue et al (1998) [30], United States	Review and triage of specialist cases (HIV, cardiology, and oral surgery) by videoconference	13	Net saving of $22 per consultation using telemedicine.	Not reported	Main saving is from saved transport
McCue et al (1997) [31], United States	Review and triage of specialist cases (HIV, cardiology, and oral surgery) by videoconference	11	Telehealth was cost saving realizing total service cost saving of $24,352 over the 7-month study period (~$21,700 per annum) or cost per visit for telehealth ($430) versus conventional care ($835).	$251,995.49	Transport savings and medical cost savings

^a^CHEERS: Consolidated Health Economic Evaluation Reporting Standards.

^b^VC: video consultation.

The most common situation where telehealth reduced health system costs reported in these studies was when it offsets patient or clinician travel funded or subsidized by the health system [17,19,21,25,26,28,30,31]. Hence, savings are most likely to be realized in the public health system, as it is unusual that other service models cover patient transport costs with the exception of the Department of Veterans Affairs. It is more likely that savings will be realized when patient travel is substituted with telehealth versus when clinician travel is substituted due to the volume, that is, saved transport cost for one clinician versus saved transport costs for many patients. One of the reviewed studies found telehealth to be less expensive than subsidized patient travel but more expensive than outreach clinics (where clinicians travel to outlying areas to provide consults) [25]. Prevention of emergency transfers was another way in which telehealth could contribute to reduced travel costs [24].

Other scenarios where potential savings can be realized include remote monitoring of high-risk pregnancies, reducing the need for in-hospital monitoring [18]. Remote monitoring in lieu of in-home visits also realized savings due to saved staff travel costs, the reduced salary of home-visit nurses, and the reduced number of hospitalizations that resulted from continual monitoring [22]. Teletriage, such as when a nurse screens a patient to determine if tertiary care is necessary, was also shown to save costs by reducing the number of hospital visits [30,31]. The two services that found telehealth to be more expensive than conventional care were due to the additional salary of a patient-end clinician (eg, general practitioner) attending a specialist consultation via videoconference [32,33].

In a number of CMA studies, telehealth was found to be cost saving as compared with standard care models only after a certain caseload was exceeded, known as a threshold or break-even point [19,21,24-26,28]. The break-even point was when the initial investment (typically in equipment setup and staff training) was offset by realized savings later on. Many studies calculated a break-even point; however, only one study reported the payback period [17]. The payback period ranged from near immediate to 9 years after implementation [34]. The payback period could be reduced if the activity was higher and the service had the capacity for increased activity [23,31]. Cost savings for telehealth have been slow to materialize for many early adopters due to the time lag between capital investments and the broader adoption of telehealth [35]. The marginal costs of a teleconsultation were found to be less than the marginal costs of conventional consultations, indicating that telehealth is likely to benefit from economies of scale [21].

### CEA

**Figure 3:**
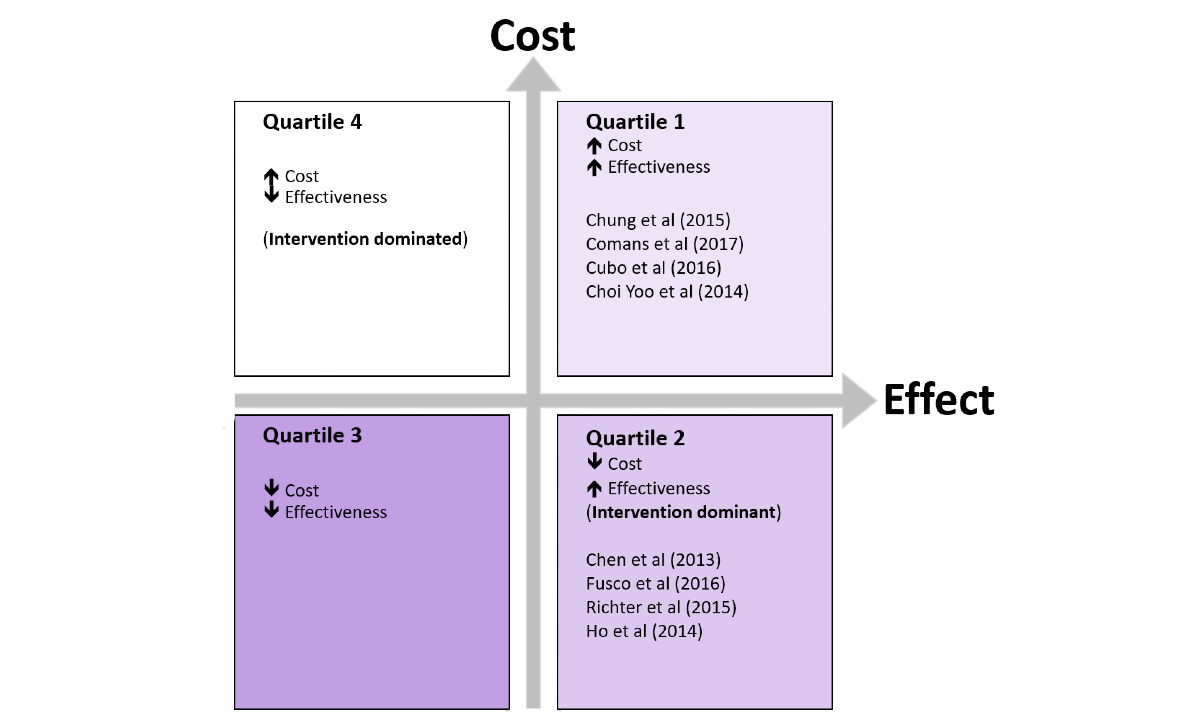
Cost-effectiveness studies mapped on cost-effectiveness plane.

A total of 8 cost-effectiveness studies from the past 5 years that reported costs from the perspective of the health system were identified [36-43]. Of the 8 studies, 4 (50%) were in quartile 2 of the cost-effectiveness plane (Figures 2 and 3), meaning they demonstrated cost savings and an increased or equivalent clinical effectiveness for telehealth compared with conventional in-person care. These are also known as dominant strategies and are recommended for implementation. Two of these studies reported results from a remote monitoring intervention for heart failure [36,42]. The remaining studies (4/8, 50%) were in quartile 1 of the cost-effectiveness plane, meaning increased clinical effectiveness for increased costs, requiring a value judgment to be made.

Moreover, 3 of the 4 cost-effectiveness studies in quartile 1 scored highly on quality as assessed using the CHEERS checklist [15], indicating comprehensive reporting of study results.

The telehealth services that were shown to reduce direct health system costs and be equally or more effective than their comparators were in smoking cessation [43], cardiovascular remote monitoring [36,42], and physiotherapy telerehabilitation after orthopedic surgery [41] (Table 3). Telehealth interventions for cardiovascular remote monitoring [36,42] and postdischarge monitoring for neonates [44] have been shown to reduce hospital admissions, readmissions, and emergency department (ED) presentations, which have the potential to reduce overall costs to the health system [36,42,44]. In these studies, hospital events were used as a measure of the effectiveness of the telehealth intervention. The premise being that preventing hospital events would reduce costs for the health system if it was translated into a dollar value.

**Table 3:** Summary of cost-effectiveness studies that demonstrated lower health system costs in the telehealth model.

Reference	Telehealth modality and clinical focus	CHEERS^a^ score	Effect measure	Effect improvement with telehealth?	Reason for lower cost in the telehealth group	Payback period
Chen et al (2013) [36], Taiwan	Remote biometric monitoring of patients with cardiovascular disease; out-of-range values trigger contact via phone from the clinical unit.	13	Hospital event rate	Yes	Reduced hospitalization, length of stay, and general medical costs to the health system when compared with similar patients without clinical monitoring and support.	Not calculable
Ho et al (2014) [42], Taiwan	Remote biometric monitoring of patients with cardiovascular disease; out-of-range values trigger contact via phone from the clinical unit.	21	Hospital event rate	Yes	Reduced hospitalization, length of stay, and general medical costs to the health system when compared with similar patients without clinical monitoring and support.	<1 year; however, due to the ongoing cost of remote monitoring, savings would need to continue at the same rate.
Fusco et al (2016) [41], Italy	Physiotherapy rehabilitation sessions delivered via videoconference to patients after orthopedic surgery.	24	Range of motion for relevant joints	Yes	Cost savings primarily due to reduced need for ambulatory government-funded travel when compared with in-person physiotherapy.	Not calculable
Richter et al (2015) [43], United States	Videoconference counselling sessions to support smoking cessation provided by primary care clinics.	17	Smoking cessation abstinence at 12 months	Equivalent	Compared with counselling provided over the phone, videoconference sessions were shorter and therefore cost less in staff wages.	Not calculable

^a^CHEERS: Consolidated Health Economic Evaluation Reporting Standards.

CEA studies use measures of effectiveness that reflect expected outcomes from the intervention. Many of these effects represent positive health gains and potentially medium- to long-term cost savings for the health system, such as avoided treatment of smoking-related diseases. However, the studies in which these effects have been demonstrated have not valued these gains in terms of cost.

### CUA

**Figure 4:**
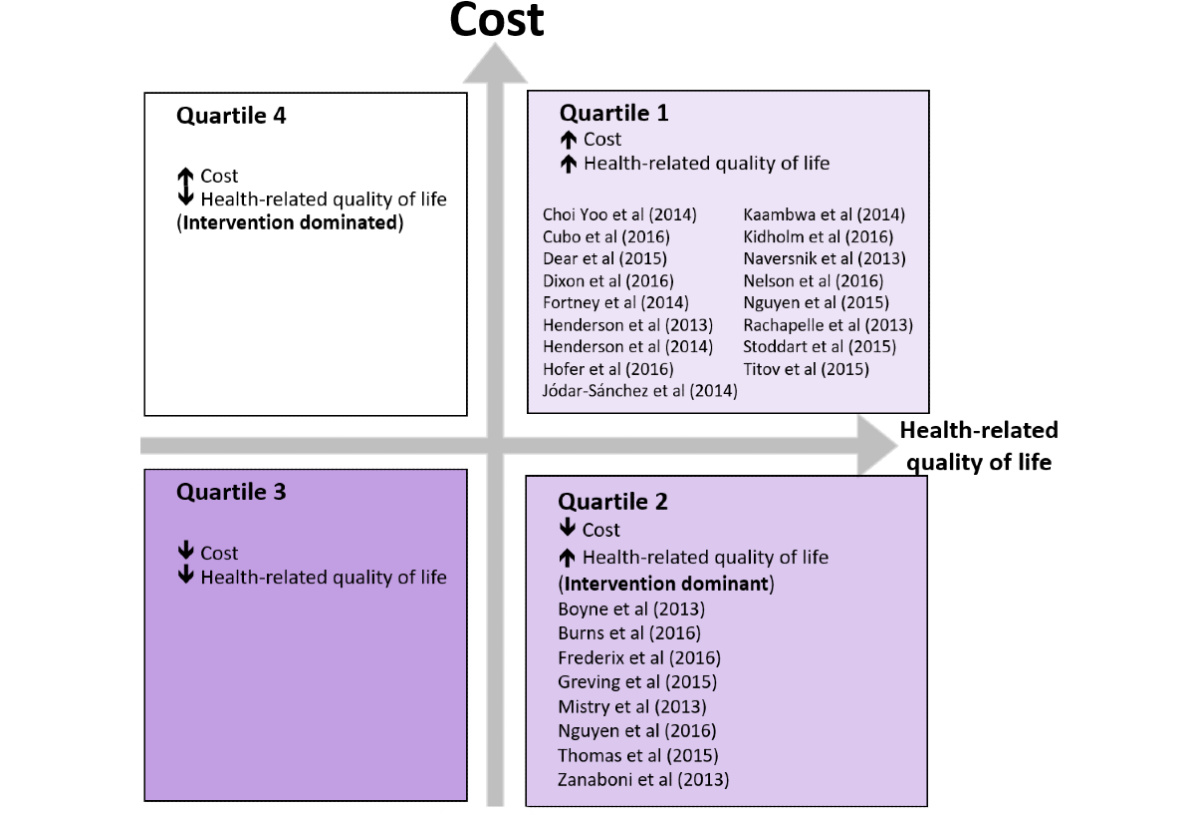
Cost-utility studies mapped on cost-effectiveness plane.

A total of 25 cost-utility studies from the past 5 years that reported costs from the perspective of the health system and changes in health-related quality of life (HRQoL) were identified [37,40,45-65]. Of these 25 studies, 8 (32%) studies were in quartile 2 of the health economics plane (Figures 2 and 4), as they demonstrated costs savings and increased or equivalent changes in effect as measured by HRQoL [45,47,49,50,57,60,65,66].

**Figure 5:**
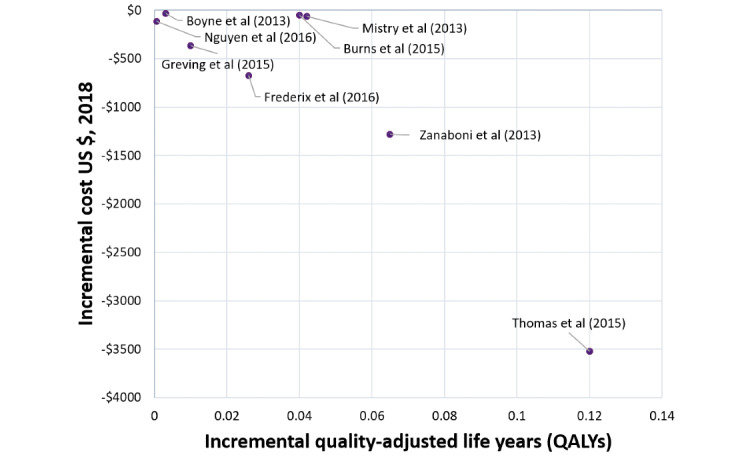
Quartile 2 incremental cost-utility values in 2019 US$.

Telehealth interventions that save money and increase quality-adjusted life years (QALYs) should be considered for implementation. As shown in Figure 5, studies in the lower right quadrant of the plane (Q2) represent telehealth interventions that, when compared with usual care, represent a decrease in health care costs and a gain in QALYs. Each of these studies describes an intervention that, if implemented, could potentially save money for the health system.

The remaining studies (17.25, 68%) represent telehealth interventions that, when compared with usual care, represent an increase in health care costs and a gain in QALYs (quartile 1; Figure 4). Therefore, these interventions would only be implemented by decision makers if they were willing to pay for the relative increase in HRQoL.

All the cost-utility studies scored highly on quality as assessed using the CHEERS checklist [15], indicating the comprehensive reporting of study results.

An incidental finding of the review was that most telehealth interventions examined provided a small marginal improvement in the number of QALYs [37,40,45,46,48-68]. This small marginal change is likely due to the sensitivity of the instruments used to measure the quality of life and their ability to respond to the changes resulting from changing the service delivery model. Increases in QALYs for telehealth interventions compared with usual care were between 0.0006 and 0.12, irrespective of the clinical area or telehealth intervention type [37,40,45,46,48-68]. This is below what is considered clinically meaningful. Studies conducted from a health system perspective reported cost savings between US $32 and US $3523. All studies were located in the dominant quartile, demonstrating that the studies that reduced costs for the health system also demonstrated either equal or increased QALYs (Figure 5; Table 4).

**Table 4:** Cost-utility analysis articles demonstrating lower cost from the perspective of the health system.

Reference	Telehealth modality and clinical focus	CHEERS^a^ score	Cost (telehealth minus usual)	Utility (telehealth minus usual)	Cost (2019 US $)	Health-related quality of life tool	Reason for lower cost in the telehealth group	Payback period
Boyne et al (2013) [45], Netherlands	In-home remote patient monitoring by a nurse. Patient’s response to clinical questions aimed at identifying exacerbation of heart failure.	22	−31 (US $, 2018)	0.0031	−31.71	EQ5D^b^	Reduction in in-person appointments and reduction in the use of physiotherapy services.	Not calculable
Frederix et al (2016) [49], Belgium	Remote monitoring for cardiovascular disease. Patients wear an accelerometer and receive feedback on their activity via email or SMS.	20	−564.4 (Euro, 2015)	0.026	−676.79	EQ5D	Reduction in rehospitalization costs.	Not calculable
Greving et al (2015) [50], Netherlands	Remote monitoring of vascular disease using patient-collected biometric information, with feedback from a remote nurse monitoring their data.	22	−219 (Euro, 2009)	0.01	−366.54	EQ5D	Reduction in paramedic support and hospital admissions.	Not calculable
Mistry et al (2013) [57], United Kingdom	Prenatal screening for congenital heart disease: store-and-forward images and videoconference consultations	23	−30 (UK £, 2009-10)	0.042	−60.1	Multiple literature sources	Economies of scale in telehealth screening compared with in-person screening.	Not calculable
Nguyen et al (2016) [60], Singapore	Store-and-forward diabetic retinopathy screening. Images captured by a nurse reviewed off-site, and the report is sent to the doctor.	20	−144 (Singapore $, not specified)	0.0006	−114.65	Time trade off	Centralized image examination was lower cost when compared with distributed image examination. Additionally, the triage process reduced unnecessary referrals for appointments and procedures.	Not calculable
Thomas et al (2015) [66], United Kingdom	Store-and-forward ophthalmic images for glaucoma screening.	22	−3569.88 (Can $, 2014)	0.12	−3523.28	Literature sources [69]	Reduction in travel and associated costs associated with travel (direct costs and staff time) and staff wages due to shorter appointments.	1-2 years for one site (saving per patient is $3570 and current annual workload is 300 patients).
Zanaboni et al (2013) [65], Italy	Remote monitoring of biometric data from an implanted device to identify heart failure exacerbations.	19	−888.1 (Euro, 2010)	0.065	−1280.46	EQ-5D^b^	Substituting in-person clinic visits with lower cost virtual consults, and reduction in the emergency department and urgent clinic visits.	1-2 years
Burns et al (2016) [47], Australia	Videoconference speech pathology for rural patients with head and neck cancer.	17	−59 (Aus $, 2015)	Equal	−48.94	Assessment of Quality of Life Instrument (4 dimensions)	Reduction in travel and associated costs and staff wages due to shorter appointments.	7-8 years for one site (saving per patient is $59 and current annual workload is 82 patients) plus 30 patients on average to cover annual levy.

^a^CHEERS: Consolidated Health Economic Evaluation Reporting Standards

^b^EQ5D: EuroQol five dimensions

### Focus Group

The expert panel focus group ran for 1 hour, and 7 experts attended from 5 institutions. Two further experts from 2 different organizations were unable to attend and provided input via email or telephone instead. They covered telehealth expertise in academic research, health service provision, and economics. Seven experts who were contacted did not respond. The videoconference session was recorded so that the investigators could review the comments during the writing of this report.

At the conclusion of the expert panel, the telehealth domains where health system costs could potentially be reduced were finalized. These domains are listed in Table 5.

**Table 5:** Domains identified from expert feedback.

Domain	Description	Level of evidence [16]
Productivity gains	Optimization of staff time leading to productivity gains.	Level II-IV
Secondary care resource use	There is potential for telehealth to reduce secondary care resource (eg, emergency department presentation, hospitalization, and medical imaging) use with associated costs savings.	Level I-III
Alternative funding models	The commercialization of telehealth has resulted in direct-to-consumer models of care where patients pay for their services directly, rather than accessing subsidized care.	Evidence in this emerging field does not map to any NHMRC^a^ levels
Telementoring	Telementoring of primary care can increase the skill level of clinicians, thereby reducing future referrals to specialists for similar cases.	Evidence in this emerging field does not map to any NHMRC levels

^a^NHMRC: National Health and Medical Research Council.

### Productivity Gains

The first domain identified by the expert focus group was productivity gains, which is when the productivity of a system is increased, cost savings can be realized due to the increased capacity of the system. In the case of telehealth, it is possible that a greater volume of patients can be managed with similar resources, which increases the productivity of the health system and reduces the marginal cost per patient. Evidence of increasing physician productivity with the use of telehealth exists in various other forms, including case control, RCT, CMA, and case series. Telehealth has the potential to increase health system productivity through a number of mechanisms, including reducing travel, reducing consultation time, and substituting for in-person service modalities.

#### Reduction in Travel

When telehealth reduces or eliminates clinician travel time because service delivery occurs through videoconference, the clinicians’ productivity is increased because they can see more patients in the same time frame. One program in the United States that implemented a videoconference telehealth model to replace home visits found that nurses’ caseload capacity more than doubled and over 14 months, 43,560 driving minutes were saved [70]. Similarly, an economic evaluation of The Northern Health Authority in Canada found that telehealth sessions replacing in-person sessions saved the health service Can $65,520 (US $49,584.14) in annual travel costs associated with clinical sessions [71].

#### Change in Consultation Time

When in-person consultations are substituted for a video consultation of a shorter duration, the clinic has an increased capacity to see more patients in the same amount of time.

Studies that compared videoconference consultation time with in-person consultation time reported disparate results, finding that videoconference was less time efficient, equally time efficient, and more time efficient. One study reported that teleconsultations may take more time than in-person appointments in cases such as assessing injuries by video as opposed to on-site [72]. Four studies reported that videoconference was equally as time efficient as in-person consultations. These included studies reporting results from studies examining services in dermatology [73], prostate cancer [74], pulmonary medicine [75], and orthopedics [76]. However, there is also evidence to suggest that videoconference consultations were more time efficient, as shown in a dermatology service from Norway [76,77] and a diabetes, antenatal, and cancer care service by Greenhalgh et al [77,78]. The small gains in clinician efficiency may be offset by increased administrative overhead associated with telehealth compared with in-person consultations [79].

#### Consultation Mode Substitution

If in-person consultations are substituted for alternate consultation modalities such as asynchronous consultations (store-and-forward or virtual), productivity is often increased as more patients are able to be managed simultaneously [5,27,80-83]. Asynchronous consultations are when the patient and clinician are not localized to the same time point. Asynchronous consultations, therefore, represent a mechanism by which the overall productivity of the health system can be improved. For instance, a specialist may be able to review clinical notes and images for a large group of patients in lesser time than it would take them to see all the patients in person [29]. Such services include dermatology consultations where either a patient or primary carer sends clinical information and dermoscopic or regular images for review; patients are then either returned to their primary care, discharged from care, or scheduled for an in-person dermatologist appointment [5,80,81,84]. Consultations are typically quicker when they are asynchronous, for example, one study found that an asynchronous dermatology consultation takes 4 min, which is one tenth of the time for a traditional consultation [85]. This increases the system throughput and can optimize both waitlists and patient prioritization. Similarly, a case-control study showed that clinicians who used a web-based messaging platform to manage patients had a 10% increase in productivity compared with those who conducted in-person consultations and followed up with phone calls [86]. The increase in productivity resulted in an additional 2.54 patients per day being seen, which in a fixed funding model would lead to a reduction in marginal cost per patient.

A study by Liddy et al [82] demonstrated that when community clinicians could send asynchronous consultations to specialists, compared with the previous system where 50% of these cases would have resulted in the referral of the patient for an in-person consultation, only 18% of cases required an in-person consultation. Another recent study showed that 68% of in-person appointments were unnecessary when an asynchronous teledermatology triage model was used [86,87].

#### Failure to Attend

When patients fail to attend appointments, it costs the health system money by increasing the marginal cost of all appointments in that service. Additionally, it represents an opportunity cost as an alternative patient has to forego an appointment. One study postulated that when patients are treated by primary care providers in their own community, the chance of them missing the appointment may be less likely than if they were traveling to a hub site further from where they live [88].

#### Payment Model

The reimbursement model needs to be considered when examining the reported productivity increases, as the marginal cost per patient will only decrease if the service provision costs remain constant as patient volume increases. A more productive clinician who is able to manage a larger number of patients will increase the cost to the health system under activity-based funding, fee-for-service, or a capitation reimbursement model. However, when clinician costs are fixed (eg, salaried), increased productivity will reduce the marginal cost per patient.

#### Summary

Telehealth can increase clinician productivity, thereby increasing the volume of patients that can be managed by a health service. Assuming fixed costs, this can result in a reduced marginal cost per patient overall. Telehealth can enable a clinician to convert travel time to clinical time, thereby improving productivity. However, when a clinician does not have to travel, substituting in-person consultations with video consultations is unlikely to have a major impact on consultation time and resultant productivity and savings. Furthermore, the increased administrative overhead for scheduling video consultations may counteract any gains. Increased productivity is more likely to be achieved in store-and-forward and virtual consultations. Realizing savings from productivity gains is dependent on the funding model. Increased productivity under activity-based funding, fee-for-service, or a capitation reimbursement model will increase the cost for the health system.

### Secondary Care Resource Use

Secondary care involves services provided by specialist or tertiary centers and includes hospital admissions, specialist outpatient visits, and ED presentations. Avoidance of secondary care in favor of other methods of care has the potential to reduce health system costs [89]. The evidence was from RCTs, case-control prospective and observational studies, and reviews of health services. Most of the studies, except the CMA conducted by Pare et al [22], reported economic findings as a secondary result. This resulted in moderate quality studies examining changes in secondary care usage related to telehealth, but a level of extrapolation from the results was needed to interpret cost savings. The use of telehealth to reduce secondary care may be realized through a number of scenarios, including remote monitoring, hospital avoidance, and triage.

#### Remote Monitoring

Remote monitoring, or telemonitoring, is an established modality of telehealth where patients are monitored from a distance. Remote monitoring is used most often to monitor chronic diseases (eg, hypertension, cardiac disease, pulmonary disease, and diabetes). Remote monitoring involves the continual in-home recording of targeted biometric readings (eg, blood pressure, glucose levels, weight, and spirometry) and in some services, patient-reported measures (eg, level of breathlessness). The readings are subsequently transmitted to a clinician for review. Remote monitoring may be performed in conjunction with in-person consults [7] or video or audio conference consults [90]. The aim of remote monitoring is the early detection and management of exacerbations, which may obviate an ED presentation or hospital admission.

Findings on secondary care usage resulting from remote monitoring usage are mixed. Telemonitoring in France, the United Kingdom, the United States, and Australia have demonstrated a reduction in hospitalization resulting directly from the use of remote monitoring compared with patients who were not monitored remotely [91-94]. In the UK trial, remote monitoring of patients with chronic disease (eg chronic obstructive pulmonary disorder, diabetes, or heart failure) was associated with an overall reduction in hospital admissions [93]. These results were consistent at three trial sites using a variety of remote monitoring technologies, such as pulse oximeters, glucometers, and weighing scales [93]. Similarly, in the United States, when patient vital signs were transmitted to a nurse for review and intervention, rates of acute care hospitalization (1.7 vs 4.4 per 1000 home health days) and ED presentations (1.9 vs 5.3 per 1000 patient days) reduced [94]. In Australia, the study by Celler et al [92] found that when using remote monitoring, clinicians were able to predict and avoid 53% of admissions by conducting a low-cost intervention in a timely manner. Remote monitoring has been shown to reduce not only a patient’s presentation to the hospital but also their length of stay once admitted. This may be due to the confidence remote monitoring gives clinicians, that when they discharge a patient, the patient is still under observation should any acute needs arise. For example, a hospital in Belgium monitored high-risk mothers at home instead of keeping them in the hospital [18]. There are, however, some studies that demonstrate in certain scenarios telemonitoring does not reduce secondary care use but, in fact, can increase secondary care usage [83,95].

When reduced secondary care usage is achieved, it would logically convert into the reduced cost for the health system. However, the economic analysis of remote monitoring does not conclusively report savings. Some studies report significant cost savings [18,22,90,96]. Other studies have found very small savings. Lew et al [97] found that for some patient groups, admission costs only reduced from US $10,835 to US $10,678. In the Whole System Demonstrator trial, cost savings amounted to a modest £188 per person per year [93]. Other studies report that remote monitoring resulted in equivalent or greater costs [97,98]. Many economic analyses of remote monitoring only report direct health care costs and do not report overall program costs such as amortization of equipment costs or the cost of running the service [7]. Other contextual factors may also influence the findings. For example, monitoring a single vital sign is less costly than monitoring multiple signs, and remote monitoring of hypertension and congestive cardiac failure is less costly than remote monitoring of respiratory diseases [7].

#### Hospital Avoidance

Telehealth can be used to facilitate hospital avoidance, which can potentially reduce costs, particularly in reducing ED presentations. Emergency Medical Services in Houston, United States, implemented a system where after an ambulance was dispatched and before the patient was transported to the ED, they conducted a videoconference with a physician [99]. Where appropriate, the patient was directed to primary care, the ED via a taxi or personal travel means or via ambulance as necessary [99]. Transports to the ED by ambulance were significantly reduced by over 50%, and the team was back in service for the next call 44 min faster [99]. A similar service in the context of residential aged care reported that the use of telehealth before transportation reduced ED presentations by 28% [100]. However, neither of these studies quantified savings.

#### Triage

Similar to findings (previously described) from the economic analyses reviewed, a recent review identified that teletriage could reduce a substantial number of unnecessary specialist outpatient appointments. The review found that for dermatology, the reported rate of avoided in-person appointments ranged from 38% to 88% and for ophthalmology ranged from 16% to 48%. Single studies for ear, nose, and throat and vascular surgery/wound care reported an 89% and 18% reduction in in-person appointments, respectively [101]. However, no study has quantified the potential cost savings.

#### Summary

Telehealth appears to have the potential to reduce secondary health care usage. However, while many studies demonstrate a reduction in secondary care, there are limited studies that quantify cost savings for the health system payer. Cost analyses do not always consider the overall costs of telehealth interventions and instead only compare the costs associated with direct health care utilization. A more accurate assessment would include program costs such as amortization of equipment costs or the cost of running the service. The use of telehealth for triage can reduce unnecessary specialist outpatient appointments. Although this would logically reduce costs for a health care provider, no study has quantified cost savings.

### Alternative Funding Models

Direct-to-consumer telehealth services are often funded by consumer payments. User pays funding models can potentially reduce costs, particularly if they substitute for government-funded or government-subsidized health services. Hence, savings to the health system are based on the assumption that individuals who access direct-to-consumer services would have otherwise accessed an equivalent health system service if the telehealth option was unavailable to them. In Australia, very few direct-to-consumer services are eligible for reimbursement from the Medicare Benefits Scheme. Consumers accessing these services are required to pay the full service fee as an out-of-pocket cost. This leads to cost savings for the government when consumers access these services instead of Medicare-funded services. Users may be willing to pay a higher out-of-pocket cost for these services because they offer elements that they value, for instance, either convenience or timely specialist access. However, due to the convenience of telehealth services, there is a risk that direct-to-consumer services may not reduce health system usage, but rather may increase overall health service utilization and costs to the health system [102]. That is, individuals who would not normally use health services may begin accessing telehealth services because of the convenience provided by the direct-to-consumer model. When these individuals are referred to a health system service provider or given a Pharmaceutical Benefits Scheme reimbursed prescription, they increase service utilization and costs to the overall health system.

There is limited academic literature that reports on savings to the health system from direct-to-consumer telehealth. Examples of commercial direct-to-consumer telehealth models include Qoctor (previously called DrSicknote), which is an Australian web-based general practitioner service. Qoctor provides a range of general practitioner services through their website, including medical certificates, prescriptions, and specialist referral letters. In January 2019, Qoctor reported $1,040,566 in saved costs to Medicare to date by diverting appropriate patients from standard general practitioner clinics [103]. A further example is iDoc24, where consumers capture images of skin lesions (eg, a mole), transmit the image to a dermatologist, and receive a diagnosis within 24 hours [104].

#### Summary

At this stage, it is difficult to quantify the cost outcomes of funding models where a consumer pays out of pocket for a commercial telehealth service. Most information is reported by the companies themselves and not through research studies. Assuming that individuals who choose to access commercial telehealth services would have instead accessed government-funded services, commercial services can reduce health system costs. As these funding models are still new, it is difficult to quantify the effect or anticipate all consequences.

### Telementoring

Deferring treatment to less qualified and, therefore, less expensive staff could potentially result in reduced health system costs. This can be facilitated by telementoring. Arguably, the most well-known telementoring program is the Project Extension for Community Healthcare Outcomes (Project ECHO), a model that was started in the United States but has been adopted and practiced internationally [105]. Using this model, primary care staff are upskilled using videoconference sessions with specialists in the form of weekly telementoring sessions. During these sessions, primary care providers can present cases and receive specialist advice on diagnosis, management, and treatment for their patients.

Although telementoring was identified by the focus group as a way to potentially reduce costs, limited evidence was found to support this view. A pre- and poststudy found that telementoring in the context of geriatric mental health resulted in a small reduction in per-patient cost when medication, specialist outpatient visits, hospitalization, and ED visits were quantified from insurance claim data [106]. Although overall costs were reduced, a subanalysis did reveal increased costs resulting from an increase in antipsychotic medication prescriptions. The time horizon for reported cost reduction was 6 months. The cost of setting up and running the telementoring was not considered in this analysis.

Project ECHO, a program that provides telementoring for primary care physicians, for the management of hepatitis C, was found to be cost-effective with an incremental cost-effectiveness ratio (ICER) of US $10,351 per QALY [107]. This would indicate that telementoring for hepatitis C will increase costs to the health system but has the potential to increase population-related quality of life.

From another perspective, savings to the health system as a result of telementoring may be realized in the retention of staff and patients at remote medical practices [88].

#### Summary

There is limited evidence due to only a very small number of studies analyzing the costs of telementoring. Telementoring can potentially reduce health system costs in both the short term and over a longer time horizon. However, at present, evidence to support this is lacking.

## Discussion

### Principal Findings

Telehealth was shown to reduce costs to the health system in the short to medium term in 53% of CMA, 50% of CEA, and 32% of CUA studies reviewed. The predominant reason for reduced costs was when the health system funded travel and either patient or clinician travel was reduced or avoided. In the remaining studies (not reviewed in detail here), telehealth increased costs but was also shown to improve care [37-40,42-44,46,48,51,52,54-56,58,59,61-64]. For example, evidence indicates that remote patient monitoring is currently a poor cost minimizer; however, it is very effective for improving overall health and reducing morbidity and hospitalization.

The models of the care and the contexts in which telehealth is used are heterogeneous. The question as to whether telehealth decreases the cost of health care delivery is complex, as are all economic evaluations in telehealth [7]. There are many compounding factors to consider, for example, the modality (real-time videoconferencing, remote patient monitoring, and store-and-forward) of telehealth used or the way telehealth consultations are remunerated. In Australia, Medicare reimburses provider telehealth consultations at 150% of an equivalent in-person consultation [108]. Furthermore, reimbursement for telehealth in Australia under Medicare and activity-based funding have both provider and patient-end payments, automatically making telehealth more expensive than conventional care. Although in the United States, state-by-state reimbursement under Medicaid means a telehealth consultation may be reimbursed at a lower or equal rate to an equivalent in-person consultation.

By improving accessibility, telehealth may also increase the cost of providing health care as populations served by traditional models of care who had limited or no access to care can now access services. Further, by improving the convenience of access, there is potential for excess use [109,110]. Furthermore, the potential for telehealth to become adjunctive to traditional in-person care (rather than substitutive) also increases the potential for increasing use and associated costs.

Telehealth is not implemented at scale in some jurisdictions (eg, Australia) [79,111], which may impact the ability to reduce costs. The marginal cost or average cost of a telehealth consultation was found to be less than an equivalent in-person consultation in a number of studies [19,32]. However, costs of running the telehealth service remained higher than the equivalent in-person service until a threshold number of consultations was reached. A threshold number of examinations required is necessary to counteract the implementation costs of telehealth, including technology and project costs. Telehealth technology costs are decreasing. Video conference equipment costs are reducing with a move from room-based systems to consumer videoconferencing platforms. A similar cost decrease of remote monitoring equipment and resultant cost decrease in remote monitoring costs per patient per year has also been reported [7]. Future studies may find a reduction in both the threshold number of examinations and the payback period resulting from a decrease in technology costs.

### Implications for Practice

Many telehealth services do not result in short- to medium-term savings. For this reason, health services considering implementing telehealth should be motivated by the benefits of telehealth other than cost reduction and recognize that implementing telehealth will require recurrent investment costs.

Asynchronous consultations are likely to reduce consultation time compared with equivalent in-person consultations, thereby improving clinician productivity. There is potential to increase the cost to the health system under fee-for-service models; hence, reimbursement for consultations where the patient is not present needs to account for increased productivity. One such example is a recent application from the Australasian College of Dermatologists for Medicare funding for store-and-forward dermatology, which sought a lower reimbursement than in-person consultations [112].

### Implications for Research

This study has identified a number of opportunities for future research. Many of the reviewed economic analyses of telehealth are older than 10 years. Contemporary economic analysis of telehealth is needed to consider changes in technology costs. Our study has also identified a gap in economic analyses related to telementoring. Furthermore, the use of telehealth to triage referrals in dermatology, wound care, otolaryngology, and ophthalmology can be effective in reducing unnecessary specialist outpatient appointments; however, there are no published cost analyses. Additionally, the use of remote patient monitoring has been shown to reduce costs and increase costs in different contexts. Many remote monitoring studies did not report overall cost savings (eg, the cost of implementing and running the service). Analyses of remote monitoring services need to include overall costs rather than direct health costs alone to determine if remote patient monitoring reduces system costs.

### Limitations

Positive reporting bias is likely to result in an increased number of studies reporting cost-effectiveness and cost-minimization of telehealth. Savings on travel resulting from telehealth are more beneficial to the health system in countries such as Australia. The large geographical areas and a public health system that funds travel favor such contexts. For this reason, the findings of cost savings are rarely generalizable. Additionally, it is unlikely that the studies examined were statistically powered for economic findings, as it is routine practice to power for the primary clinical outcome, meaning economic outcomes may not be precise. The aim of this study was to identify short- to medium-term cost savings; therefore, a further limitation of this study is that studies examining the long-term cost impact of telehealth interventions were not included.

### Conclusions

This study aimed to determine whether telehealth has the potential to improve the sustainability of health systems by reducing costs. Telehealth was shown to reduce costs to the health system in the short to medium term in 53%, 50%, and 32% of the cost-minimization, cost-effectiveness, and cost-utility studies reviewed, respectively. The predominant reason for reduced costs was when the health system–funded travel (patient or clinician) was reduced or avoided. In the remaining studies, telehealth increased costs, albeit with improved care.

The expert focus group identified 4 areas of potential savings from telehealth: productivity gains, reductions in secondary care use, emerging alternate funding models for care provision, and savings resulting from telementoring effects. Telehealth can increase clinician productivity when it is used to convert travel time to clinical time. In terms of consultation time, there are unlikely to be productivity gains when substituting an in-person consultation with a video consultation. The use of asynchronous consultations as a substitute for in-person consultations is more likely to increase productivity by reducing consultation time. However, under activity-based funding mechanisms, there is a likelihood that these productivity gains could result in cost increases.

Mitigation of secondary care through remote patient monitoring, teletriage, and hospital avoidance has the potential to reduce costs to the provider. However, there is currently a lack of economic evidence to support this. Similarly, telementoring has scant economic evaluations to demonstrate cost savings.

Alternate funding models from telehealth systems have the potential to save the health system money in situations where the consumers pay fully out of pocket to receive services, thereby mitigating the cost to the health system. The convenience of telehealth may influence consumers to pay out-of-pocket fees. This may be considered cost-shifting as opposed to cost saving.

The available evidence has indicated that telehealth does not always reduce the cost of care from the perspective of the health system in the short to medium term. Health services considering implementing telehealth should be motivated by benefits other than cost reduction.

## Abbreviations

CEA: cost-effectiveness analysis
CHEERS: Consolidated Health Economic Evaluation Reporting Standards
CMA: cost-minimization analysis
CUA: cost-utility analysis
ED: emergency department
GDP: gross domestic product
HRQoL: health-related quality of life
PRISMA-ScR: Preferred Reporting Items for Systematic reviews and Meta-Analyses extension for Scoping Reviews
Project ECHO: Project Extension for Community Healthcare Outcomes
QALYs: quality-adjusted life years
RCTs: randomized controlled trials
